# Editorial: The transcriptional and metabolic role of Foxp3 in regulatory T cells

**DOI:** 10.3389/fimmu.2026.1796661

**Published:** 2026-02-11

**Authors:** Mehdi Benamar

**Affiliations:** Institute of Regenerative Medicine and Biotherapy, INSERM, University of Montpellier, Montpellier, France

**Keywords:** autoimmunity, chronic inflammation, FOXP3, regulatory T (Treg) cell, Treg cells metabolism

Inflammation is an indispensable feature of immune defense, yet when unchecked it becomes a driving force behind chronic disease, autoimmunity, and tissue destruction (1, 2). The immune system therefore requires dedicated mechanisms to restrain excessive responses while preserving protective immunity. At the center of this delicate immunological balance reside regulatory T cells (Tregs), a specialized lineage of CD4 T cells defined by expression of the transcription factor FOXP3, a member of the forkhead family of transcription factors (3–7). Early insights into FOXP3 established its necessity for Treg lineage commitment and stability (5, 8), with mutations in FOXP3 resulting in profound immune dysregulation and multi-organ autoimmunity (9). But beyond lineage specification, FOXP3 actively shapes the Treg transcriptome, collaborating with co-factors and chromatin regulators to fine-tune gene expression in a context-dependent manner (10–13). This flexible chromatin engagement allows Tregs to occupy distinct functional states, enabling suppression, tissue repair, and even metabolic regulation in response to local demands. Over the past 30 years, advancements in Treg biology have profoundly reshaped our understanding of immune homeostasis, revealing not merely suppressors of inflammation, but dynamic orchestrators of immunological tolerance and tissue integrity. Tregs employ a multifaceted repertoire of mechanisms to restrain pathological immune responses. These include secretion of anti-inflammatory cytokines such as IL-10, IL-35 and TGF-β, modulation of antigen-presenting cell function, metabolic competition for IL-2, and direct cell contact-dependent suppression (14). They communicate with cells of both innate and adaptive immunity, tempering effector responses while preserving capacity for protective host defense. Importantly, the phenotypic and functional heterogeneity within Treg populations, shaped by tissue-specific cues and molecular signatures, has emerged as a critical determinant of their regulatory capacity in distinct inflammatory environment (15–21). Treg cells subsets distinguished by transcriptional programs, surface markers, and tissue residency profiles can exert specialized roles in contexts ranging from mucosal tolerance to systemic autoimmune regulation (16). This heterogeneity presents both an intellectual challenge and an extraordinary therapeutic opportunity: understanding how distinct Treg subsets develop, navigate inflamed tissues, and reprogram local immune networks promises new avenues for precision immunotherapy.

Central to this remarkable functional repertoire is FOXP3 whose sustained expression defines Treg identity and function. Concurrent with its transcriptional influence, Foxp3 orchestrates metabolic programs that are essential to Treg function and resilience. Unlike conventional effector T cells that rely heavily on glycolysis to fuel rapid proliferation, Tregs preferentially engage oxidative phosphorylation (OXPHOS) and fatty acid oxidation, metabolic pathways that support their suppressive capacity and long-term survival (22). Foxp3 actively represses glycolytic genes while promoting mitochondrial fitness, enabling Tregs to maintain function even in nutrient-challenged or inflammatory environments such as tumors, ischemic tissues, or inflamed mucosa (22). This metabolic specialization is not merely a correlate of identity but a mechanistic pillar of Treg action: dysregulated metabolism compromises suppressive function and can precipitate inflammatory disease. The metabolic landscape encountered by Tregs varies widely across tissues. Tregs resident in these niches not only withstand low-glucose and high-lactate conditions but can exploit these environments through Foxp3-dependent metabolic reprogramming. By suppressing glycolysis and enhancing OXPHOS, Foxp3 equips Tregs with a metabolic edge in hostile microenvironments, a feature that likely contributes to their dominant role in tissue homeostasis and regulation (23, 24).

This Research Topic brings together a rich collection of articles that illuminate the transcriptional and metabolic dimensions of Foxp3 biology. From mechanistic perspectives on how Foxp3 collaborates with transcriptional networks to emerging insights into metabolic heterogeneity and flexibility. Articles delve into how alternative splicing of Foxp3 impacts metabolic pathways, how Foxp3 partner proteins modify Treg fate and function, and how metabolic cues intersect with epigenetics to influence immune outcomes in autoimmunity, infection, and cancer. In summary, Foxp3 sits at a strategic crossroads, linking the transcriptional architecture of Tregs with their metabolic phenotype. Deciphering this linkage is not only of fundamental scientific interest but also of profound clinical importance in diseases where immune balance is lost. The work showcased in this Research Topic represents a significant stride toward that understanding, and promises to catalyze new discoveries at the intersection of gene regulation and immunometabolism. Indeed, harnessing Tregs as therapeutic agents represents one of modern immunology’s most exciting frontiers. Early clinical trials have demonstrated the safety of Treg cell therapies, and ongoing studies are increasingly focused on efficacy, stability, and precision targeting. Genetically engineered Tregs, tailored for enhanced suppressive activity or directed to specific inflammatory antigens, hold particular promise for conditions where conventional treatments fall short. Looking forward, the challenge is to translate our expanding molecular and cellular insights into interventions that safely and effectively restore immune equilibrium. Finally, this Research Topic highlights not only how far the field has come, but also how much remains to be discovered. By continuing to unravel their complexity, we take critical steps toward therapies that could transform the management of inflammatory disease across broad therapeutic landscapes. Looking ahead, the integration of single-cell genomics, spatial transcriptomics, and metabolic profiling promises to deepen our understanding of how Foxp3 shapes Treg identity *in situ*. Such high-resolution insights will be essential to design precision therapies that calibrate immune regulation with minimal off-target effects.
